# Prevalence and risk factors of compulsory admissions in athens region: Are there any differences between psychiatric and general hospitals?

**DOI:** 10.1192/j.eurpsy.2021.1045

**Published:** 2021-08-13

**Authors:** L.E. Peppou, N. Drakonakis, S. Stylianidis

**Affiliations:** 1 Department Of Psychology, Panteion University of Social Sciences, Athens, Greece; 2 Unit Of Social Psychiatry & Psychosocial Care, University Mental Health, Neurosciences and Precision Medicine Research Institute “Costas Stefanis” (UMHRI), Athens, Greece; 3 3rd Clinic, Psychiatric Hospital of Attica, “Dafni”, Athens, Greece

**Keywords:** compulsory admissions, deinstitutionalization, involuntary hospitalizations, psychiatric reform

## Abstract

**Introduction:**

Concerns have been raised about Europe facing a reinstitutionalization process. Thus, research and policy interest in prevalence and determinants of involuntary hospitalizations has recently rekindled. In Greece, heightened rates of compulsory admissions have been partly ascribed to the incomplete psychiatric reform. Psychiatric hospitals remain the mainstay of inpatient care, as opposed to the more community-oriented psychiatric departments of general hospitals.

**Objectives:**

To investigate differences between a psychiatric and a general hospital with respect to rates and determinants of involuntary hospitalizations in Athens.

**Methods:**

All admissions in one psychiatric and one general hospital between May – September 2020 were considered. Information about patients’ socio-demographic characteristics and mental health status was garnered through clinical records and patient and physician interviews. Symptom severity was assessed with the Health of Nations Outcome Scale and diagnosis was assigned in accordance with the ICD-10 criteria.

**Results:**

A total of 600 admissions were analysed. In the general hospital, 52.5% of admissions were involuntary, as opposed to 63.1% in the psychiatric hospital (OR = 0.65, 95%CI = 0.43 – 0.97). In the general hospital, the sole risk factor for compulsory admission was aggression (OR= 3.23, 95%CI = 1.24-8.4). Interestingly, in the psychiatric hospital, sex, age, nationality, education, diagnosis and the severity of symptoms tapped by HoNOS were not found to predict involuntary status.

**Conclusions:**

In psychiatric hospitals, no patient subgroups appear to be at elevated risk of civil detention. Therefore, further research is warranted as to what drives the decision there.

